# Accurate IMRT fluence verification for prostate cancer patients using ‘in-vivo’ measured EPID images and in-room acquired kilovoltage cone-beam CT scans

**DOI:** 10.1186/1748-717X-8-211

**Published:** 2013-09-10

**Authors:** Ali SAM Ali, Maarten LP Dirkx, Ruud M Cools, Ben JM Heijmen

**Affiliations:** 1 Department of Radiation Oncology, Erasmus MC-Daniel den Hoed Cancer Center, Groene Hilledijk 301, 3075 EA, Rotterdam, The Netherlands

**Keywords:** EPID dosimetry, Treatment verification, Prostate cancer, CBCT

## Abstract

**Background:**

To investigate for prostate cancer patients the comparison of ‘in-vivo’ measured portal dose images (PDIs) with predictions based on a kilovoltage cone-beam CT scan (CBCT), acquired during the same treatment fraction, as an alternative for pre-treatment verification. For evaluation purposes, predictions were also performed using the patients’ planning CTs (pCT).

**Methods:**

To get reliable CBCT electron densities for PDI predictions, Hounsfield units from the pCT were mapped onto the CBCT, while accounting for non-rigidity in patient anatomy in an approximate way. PDI prediction accuracy was first validated for an anatomical phantom, using IMRT treatment plans of ten prostate cancer patients. Clinical performance was studied using data acquired for 50 prostate cancer patients. For each patient, 4–5 CBCTs were available, resulting in a total of 1413 evaluated images. Measured and predicted PDIs were compared using γ-analyses with 3% global dose difference and 3 mm distance to agreement as reference criteria. Moreover, the pass rate for automated PDI comparison was assessed. To quantify improvements in IMRT fluence verification accuracy results from multiple fractions were combined by generating a γ-image with values halfway the minimum and median γ values, pixel by pixel.

**Results:**

For patients, CBCT-based PDI predictions showed a high agreement with measurements, with an average percentage of rejected pixels of 1.41% only. In spite of possible intra-fraction motion and anatomy changes, this was only slightly larger than for phantom measurements (0.86%). For pCT-based predictions, the agreement deteriorated (average percentage of rejected pixels 2.98%), due to an enhanced impact of anatomy variations. For predictions based on CBCT, combination of the first 2 fractions yielded gamma results in close agreement with pre-treatment analyses (average percentage of rejected pixels 0.63% versus 0.35%, percentage of rejected beams 0.6% versus 0%). For the pCT-based approach, only combination of the first 5 fractions resulted in acceptable agreement with pre-treatment results.

**Conclusion:**

In-room acquired CBCT scans can be used for high accuracy IMRT fluence verification based on in-vivo measured EPID images. Combination of γ results for the first 2 fractions can largely compensate for small accuracy reductions, with respect to pre-treatment verification, related to intra-fraction motion and anatomy changes.

## Introduction

With the introduction of advanced treatment techniques like intensity modulated radiotherapy (IMRT) and volumetric modulated arc therapy (VMAT), the high-dose volume can generally be better conformed to the target volume, while achieving steep dose gradients towards surrounding normal tissues. As a result, the accuracy of patient positioning and dose delivery becomes more critical. To verify delivered treatment fields, dosimetric pre-treatment verification is performed prior to the first treatment fraction using films, 2D detector arrays or Electronic Portal Imaging Devices (EPIDs) in many institutes.

EPIDs have shown to be suitable for dose verification, both pre-treatment and in-vivo because of their high resolution, acquisition of digital data in 2D in a short acquisition time, and high measurement accuracy [1-10]. In our institute, for over eight years, pre-treatment EPID dosimetry is performed for all IMRT patients to compare fluence maps delivered by the linac with TPS prescriptions. For this purpose, prior to the first treatment fraction, a full treatment including gantry rotations is executed in the absence of the patient and treatment couch, while acquiring for each beam an EPID image. These images are converted into Portal Dose Images (PDIs) [10] and compared with predictions, based on the prescribed leaf sequence and monitor units (MU). For several patients, major errors were captured prior to the start of treatment [2]. Similar errors, like a malfunctioning leaf or unintended changes of treatment parameters in the record and verify system have also occurred during patient treatment, after the pre-treatment verification was successfully performed. So, in spite of the high overall reproducibility of delivered IMRT fluence maps (within 1% (1 SD), as assessed from repeated EPID measurements) [11], pre-treatment verification does not guarantee correct fluence delivery to the patient during the course of treatment. For this purpose, in-vivo dosimetry may be performed. When in-vivo PDI predictions are based on the pCT of the patient, comparison with measured PDIs may result in an unreliable estimate of the fluence delivered by the treatment unit, because of changes in patient positioning and/or patient anatomy (e.g., due to deformation, changing air gaps or weight loss). To be able to accurately derive delivered fluences irrespective of changes in patient anatomy, the Split IMRT Field Technique (SIFT) [12] was introduced in our institute. For this technique, each IMRT field is split into a low MU static field and a residual IMRT field. From the ratio of the PDIs of these fields, both being acquired in a short time interval, the fluence of the IMRT field can be derived within about 1% accuracy. The SIFT method has several limitations: (i) deviations in beam output (cGy/MU) and in open beam profile cannot be detected, because they cancel out in the ratio image, (ii) in commercially available treatment planning systems (TPS), splitting of an IMRT field into a static and residual modulated field is often not possible, and (iii) the technique is only applicable to IMRT; 3D conformal radiotherapy treatments or rotational treatments, like Rapid Arc or VMAT, cannot be verified in a similar way.

CBCTs are increasingly being used to verify and correct target set-up. In this study, we investigated the accuracy of in-vivo fluence verification by analyses based on PDIs derived from measured EPID images and predictions calculated using a CBCT acquired in the same treatment fraction. It was hypothesized that an in-room acquired CBCT would better represent the patient’s anatomy during acquisition of the EPID images than the pCT would do, allowing for an increased PDI prediction accuracy and resulting in an enhanced sensitivity for detecting fluence maps deviations. Therefore, for evaluation purposes, PDIs were also predicted using the patients’ pCTs. It was investigated whether combining in-vivo measurements performed in the first 1–5 fractions could enhance agreement between measurements and predictions, comparable to pre-treatment verification.

## Materials and methods

### Prostate IMRT, CBCT and pCT acquisition

Prostate IMRT plans, consisting of 5–7 fields, were generated with the Monaco TPS (Elekta AB) and delivered with a step-and-shoot technique on an Elekta Precise treatment unit. The beam energy was 10 MV. During the first three treatment fractions, and once in the third and sixth week of treatment, kilovoltage cone-beam projection images were acquired using a gantry-mounted XVI system (Elekta AB), CBCT images were reconstructed with a 2 mm slice distance using the XVI software. Treatment planning CT-scans (pCT) with a 3 mm slice thickness and a 2.5 mm slice distance were acquired with a Somaton Sensation Open multi-slice CT scanner (Siemens, Erlangen, Germany).

### In-vivo PDI measurement and prediction

Electronic portal images were acquired with a CCD-camera based EPID (Cablon Medical Theraview Technology, Leusden, The Netherlands). The dosimetric characteristics of this EPID have been extensively reported before [13]. A measured EPID image was converted into an absolute PDI by deconvolution with position-dependent cross-talk kernels [10,14] and normalization with a conversion factor derived from an EPID image for a symmetric 10 × 10 cm^2^ field irradiated with 100 MU.

To predict the in-vivo PDI, first a pre-treatment PDI was calculated from the prescribed incident fluence on the EPID (without patient in the beam), as derived from the planned MLC leaf sequence and the number of MU [10]. Then corrections for beam attenuation and radiation scattered in the patient (or phantom) towards the EPID were calculated based on (imaginary) equivalent homogeneous phantom (EHP) [15]. For each treatment beam, a separate EHP was calculated. These EHPs are derived from the patients’ pCT (or CBCT) and for each ray line have a polystyrene-equivalent thickness and equal distance between the centre of mass and the EPID plane. Based on a previously validated model for the treatment couch [16], its beam absorption was also accounted for in the EHP.

### Correction of CT values in CBCT images

Compared to conventional CT scanners, kilovoltage CBCT images may suffer from an increased contribution of scattered radiation, being largely dependent on the patient geometry [17]. Consequently, CT values in those CBCT images do generally not accurately represent electron densities, and the CBCT cannot directly be used for establishing an EHP to be used for prediction of in-vivo measured PDIs either. To adjust CT values, we applied a previously described method [18]. First, the CBCT was aligned to the pCT using rigid registration based on mutual information. After resampling the pCT on the grid of the CBCT, the Hounsfield units of the pCT were mapped onto the CBCT. To preserve the shape of the body outline in the CBCT scan, all pixels outside this surface were assigned the standard CT value for air (−1000 HU) and all pixels inside the body surface of the CBCT, but outside the body outline of the pCT scan, were assigned the standard CT value for water (0 HU). Similarly, to preserve the shape of air cavities in the CBCT scan, all pixels inside air cavities in the CBCT got the value −1000 HU, and pixels inside cavities in the pCT scan, but outside cavities in the CBCT, were given the value 0 HU.

### Validation of in-vivo fluence verification using phantom measurements

To validate fluence verification based on comparison of in-vivo measured PDIs with PDI predictions established with an in-room acquired CBCT, IMRT treatment plans of 10 prostate cancer patients were delivered to an anatomical phantom, representing the caudal part of the abdomen and the upper part of the pelvic, made of solid water and lung equivalent material (Gammex). For each of the in total 68 fields, an EPID image was acquired during beam delivery. For PDI prediction, EHPs were derived from the CBCT after mapping the Hounsfield units of the pCT (see above). For comparison, predictions were also performed for the pCT, and pre-treatment analyses (i.e., without phantom and treatment couch in the beam) were performed as well.

### Clinical evaluation of in-vivo fluence verification

Clinical evaluation was performed for a group of 50 consecutive radically treated prostate cancer patients. In each fraction, prior to dose delivery, the position of the prostate was verified by visualizing implanted gold markers using a crossfire of kV and MV imaging [19]. An on-line set-up correction was then applied for deviations exceeding 2 mm in vector length. From the left and the right lateral beams, a segment with 6 MU was split to be able to verify the prostate position during treatment as well [20]. When a 4 mm threshold was exceeded, a correction for the intra-fraction motion was applied. For verification purposes, in the first three treatment fractions, and once in the third and sixth week of treatment, also a CBCT was acquired after delivery of all fields. For those fractions, acquired PDIs were compared to PDIs, predicted both using the acquired CBCTs with corrected Hounsfield units (see above) and the pCT. In total 313 pre-treatment images and 1413 in-vivo dosimetry images were evaluated.

### Comparison of measured and predicted PDIs

Measured and predicted PDIs were compared using γ evaluations, applying 3% global dose difference and 3 mm distance to agreement as reference criteria. Pixels with a portal dose lower than 10% of the maximum dose inside the field were ignored. For each comparison, the mean γ and the percentage of rejected pixels, i.e., pixels with a γ value larger than 1, were derived. In addition, a previously published automatic decision scheme for comparison of PDIs was applied [2]. According to this scheme, a PDI analysis is automatically approved when the percentage of failing pixels is less than 15% and the size of clustered areas with pixels having a γ larger than 1 is smaller than 1 cm^2^. For clustered areas with sizes between 1 cm^2^ and 5 cm^2^ this also applies when in each of them the mean gamma is less than 1.5 and the maximum gamma is less than 2. When larger differences are detected, the automatic evaluation fails and a physicist needs to inspect the results visually to assess whether the observed deviations between measured and predicted PDIs are clinically relevant or not.

### Combining results from multiple fractions

To be able to distinguish systematic deviations between measured and predicted PDIs from non-clinically relevant deviations occurring in a single fraction, we applied a previously described method to combine γ results from the first n treatment fractions [21]. Taking one field at a time, corresponding γ pixel values from successive measurements were first sorted in ascending order. Then, a composite “low” γ-image [21] was derived with pixel values halfway between the minimum and the median γ value per pixel.

## Results

### Validation of in-vivo fluence verification using phantom measurements

When using the CBCT for in-vivo PDI prediction, the average mean γ for the 68 evaluated beams was 0.30 ± 0.03 (1 SD) and the average percentage of rejected pixels was as low as 0.81 ± 0.65%. As to be expected for a phantom, a *t*-test did not show any significant difference with respect to the use of the pCT for predictions, yielding an average mean γ of 0.31 ± 0.04% (*p* = 0.75) and an average percentage of rejected pixels of 0.86 ± 0.66% (*p* = 0.93)). For pre-treatment verification, deviations between measured and predicted PDIs were slightly, but significantly, smaller with an average mean γ of 0.26 ± 0.03 (*p* < 0.001) and an average percentage of rejected pixels of 0.44 ± 0.44% (*p* = <0.001). These improved results were mainly related to a higher signal-to-noise ratio in the pre-treatment images and uncertainties in the transmission model used for in-vivo dosimetry.

### Clinical evaluation of in-vivo fluence verification

**Figure 1:**
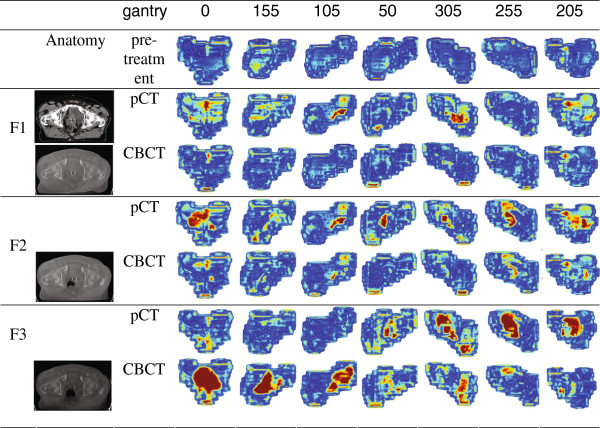
**γ evaluations of the observed differences between measured and predicted PDIs.** Three treatment fractions (F1, F2, and F3) of one patient are shown. The beams are shown in the order that they were delivered. PDI predictions were based on either CBCT or pCT. As a reference, the results from the corresponding pre-treatment measurements are depicted as well. Representative transversal CT slices from the pCT and the CBCT, acquired at the end of each fraction, are shown as well.

Figure 1 shows PDI comparisons for a patient during three treatment fractions, and the corresponding pre-treatment analyses. For all beams in the first two fractions and the last beams in the third fraction, the agreement between measured and predicted PDIs was clearly improved when using the CBCT instead of the pCT for PDI prediction. For the first beams in the third fraction, CBCT performed worse than pCT. Apparently, the CBCT, acquired at the end of treatment, did not accurately represent the patient anatomy at the start of treatment due to intra-fraction anatomy changes, while for the pCT this applied for the end of treatment.

**Figure 2:**
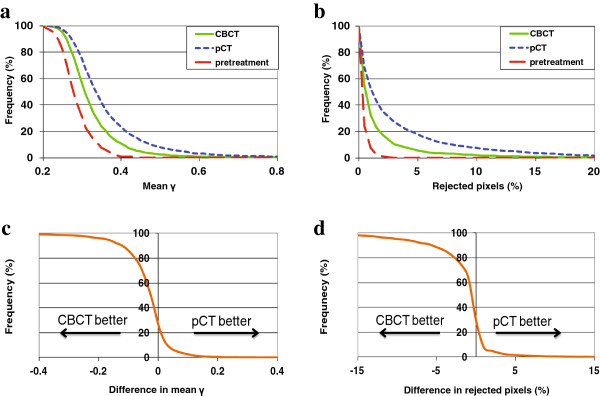
**Cumulative histograms of (a) observed mean γ values and (b) percentages of rejected pixels when comparing measured and predicted PDIs.** Prediction of in-vivo PDIs for the 1413 measured images was based on either CBCT or pCT. Results for the corresponding 313 pretreatment measurements were included as well. **(c)** and **(d)** show cumulative histograms of differences between CBCT- and pCT-based prediction.

For the 1413 evaluated in-vivo measured images, the distributions of observed mean γ values and percentages of rejected pixels are depicted in Figure 2. In more than 70% of images the mean γ and/or percentage of rejected pixels was lower for the CBCT-based PDI prediction (Figures 2c and 2d). For images with lower γ parameters for the pCT-based PDI prediction, the differences compared to CBCT were generally small. By using CBCT instead of pCT, the average mean γ reduced from 0.36 ± 0.10 to 0.32 ± 0.07 (p < 0.001). For pCT the percentage of rejected pixels was already low (2.98 ± 4.88), but with CBCT this reduced to 1.41 ± 2.78% (p < 0.001). The corresponding values for the 313 pre-treatment images were 0.28 ± 0.04 and 0.35 ± 0.41%, respectively. For pre-treatment verification, all images passed the automated PDI comparison scheme. For in-vivo measured images the success rate reduced to 95.8% and 87.9% for CBCT- and pCT-based PDI prediction, respectively.

### Combining results from multiple fractions

**Figure 3:**
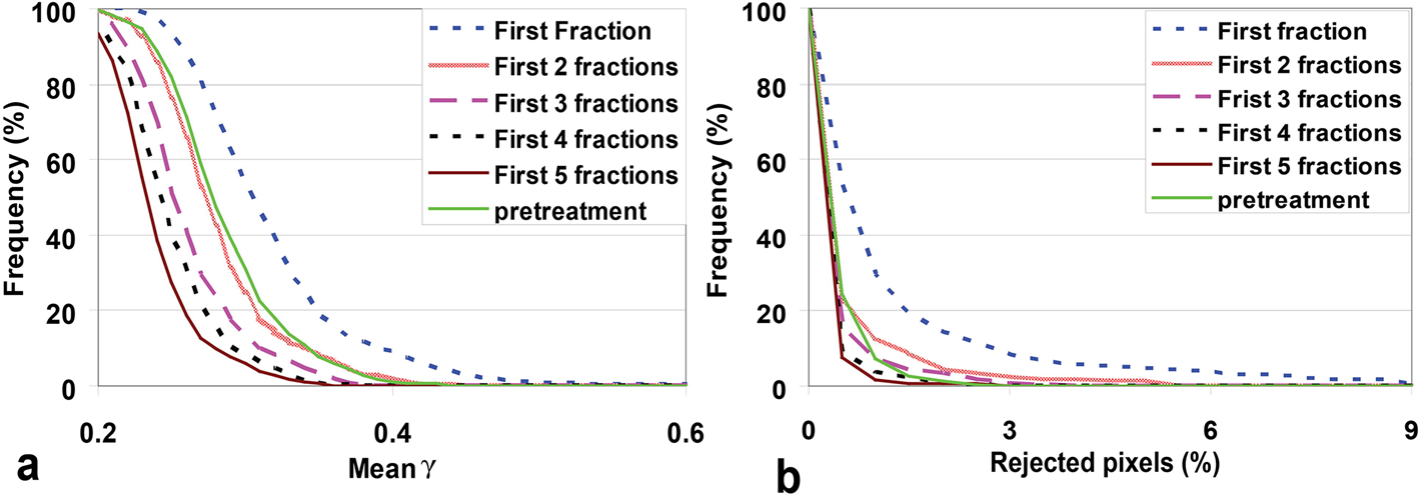
**Cumulative histograms of (a) observed mean γ values and (b) percentages of rejected pixels when comparing measured and predicted PDIs for combined fractions.** For in-vivo measured PDIs, results are shown for images acquired in the first fraction and for combinations of the first 2, 3, 4, and 5 fractions. All in-vivo PDI predictions were based on a CBCT. CBCT and in-vivo PDIs were always acquired in the same fraction.

**Figure 4:**
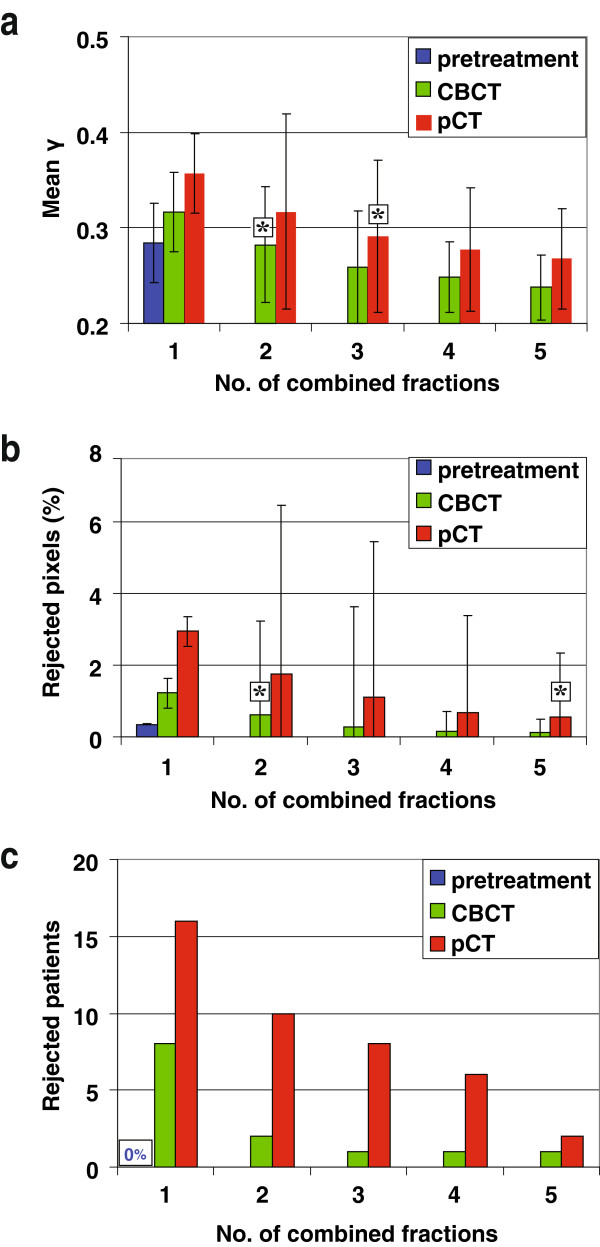
**Comparison of predicted and measured PDIs. (a)** Averages of observed mean γ values, **(b)** averages of percentages of rejected pixels, **(c)** number of patients in which one or more beams were rejected according to the applied decision scheme. For in-vivo analyses, predictions were based either on CBCT or pCT. Results are shown for only the first fraction and for combinations of the first 2, 3, 4, and 5 fractions. The bars show the spread in the observations (±1 SD). Values indicated by * are *not* statistically significant different from the pre-treatment results, as established with paired t-tests.

Figure 3 shows that combining γ images from several initial fractions could substantially reduce differences between in-vivo measurements and predictions based on CBCT. Already when combining only the first 2 fractions, the frequency distributions for the mean γ (Figure 3a) and the percentage of rejected pixels (Figure 3b) approached the corresponding pre-treatment distributions very closely. Figure 4 clearly demonstrates that PDI prediction based on CBCT resulted in smaller deviations from measurements than pCT-based prediction did. After combination of the first 2 fractions in the CBCT approach, the difference in average percentage of rejected pixels and mean gamma with pre-treatment became statistically insignificant. In contrast, for pCT-based prediction, insignificant differences in the mean gamma were obtained after combining the first 3 fractions, while for the mean gamma even 5 fractions should be combined (Figure 4a, Figure 4b). In the pre-treatment approach, all fields passed the automated PDI comparison scheme. After combination of 2 in-vivo fractions, the number of patients in which one or more beams were rejected (rejected patients) was two patients (2 images) for CBCT-based PDI predictions; for pCT-based predictions this applied to nine patients (23 images). When combining 3 fractions these numbers dropped to one patient (1 image) and eight patients (18 images), respectively. Two patients (2 images) were still rejected for predictions based on the pCT after combining 5 fractions (Figure 4c).

## Discussion

As far as we know, this is the first article describing the use of kilovoltage CBCT for prediction of in-vivo measured PDIs to verify delivered IMRT fluence map with an EPID. As expected, the agreement between measured PDIs and CBCT-based predicted PDIs was slightly better in phantom experiments than for patients. This is partly attributed to intra-fraction patient motion. For some patient fractions, the acquired CBCT did not accurately represent the patient’s anatomy for all treatment beams and hence EPID acquisitions. Fraction 3 in Figure 1 shows an example. At the start of treatment, the agreement between measured and CBCT-based predicted PDIs was clearly less good than for beams acquired near the end of the fraction, i.e., shortly before the CBCT was acquired. Apparently, the acquired CBCT did not accurately represent the patient anatomy at the start of treatment, likely due to intra-fraction anatomy changes. It should be noted that the applied on-line set-up correction procedure to account for observed inter- and intra-fraction prostate displacements, as derived from imaged implanted fiducials, could not (fully) account for these effects.

Figure 3 shows that also for in-vivo dosimetric analyses based on CBCT in one single fraction, gamma deviations between measurements and predictions are low, which allows detection of large, clinically relevant errors immediately after the first fraction. Combination of in-vivo results in multiple fractions reduces the impact of intra-fraction motion, and averages out random variations in fluence delivery that do not occur in all fractions. On the other hand, reproducible errors in fluence delivery will not diminish. Consequently, by combining multiple fractions, the sensitivity for detection of systematic errors in fluence delivery (i.e., occurring in all fractions) will increase. This approach was initially suggested by McDermott et al. [21]. For a group of 75 prostate cancer patients, they concluded that by combining in-vivo dosimetry results from 3–5 treatment fractions using ‘low’ γ images, the results were in close agreement with pre-treatment results. In this study, we also investigated the use of these low γ images, both for CBCT- and pCT-based predictions. For the pCT-based approach we found that 4–5 fractions had to be combined to approach pre-treatment results. When using CBCT, already combination of only the first 2 fractions yielded results in very close agreement with pre-treatment analyses. Obviously, even with some intra-fraction motion, CBCT scans do much better represent patient anatomy during the fractions than the pCT does and consequently a more accurate fluence verification was possible than with pCT scans.

Over three years ago a computer-controlled system is running in our department that daily checks whether treatment parameters in our record-and-verify system correspond to the parameters of the plan that was prescribed and approved by the physician [22]. With this system we also check *pre-treatment* whether a treatment plan is correctly transferred from the planning system to the record-and-verify system. In addition, a redundant monitor unit calculation is performed prior to start of treatment for all our patients. To correct for inter- and intra-fraction motion in prostate cancer patients, on-line set-up verification is performed before and during each treatment fraction [19,20]. On top of these individualized QA procedures, the IMRT performance of our linacs is checked daily with EPID measurements during the morning checks [23,24]. During the last eight years, we performed pre-treatment verification for all IMRT patients. For less than 0.3% of the patients, clinically relevant errors were detected. Because of the large number of IMRT patients, performing pretreatment verification for all of them is a workload intensive practice, involving measurements during evenings. Moreover, pre-treatment verification inherently checks profiles that are not necessarily equal to those delivered to the patient.

The results obtained in this work gave us confidence that for prostate patients treated with a large number of fractions, IMRT fluence delivery verification with pre-treatment EPID dosimetry may be replaced by in-vivo EPID dosimetry, when combined with the QA steps mentioned before (in particular the pre-treatment check on data transfer). This has the advantage that fluence delivery verification is performed while the patient is being treated with a plan that has been checked pre-treatment for data transfer errors. In practice, we verify the first 3 fractions, and also analyze the combined fractions, as described in this paper. A relevant side effect is that measurements in evenings and weekends can largely be reduced. Of course, our method can also be used as a supplement to pre-treatment verification, adding fluence verification during patient treatment. In principal, this can be done in each fraction.

Due to the high accuracy, in-vivo verification based on CBCT allows for detection of clinically significant errors with minimal user interaction after the first treatment fraction, as is evident from the gamma results and high percentage of accepted images (95.8%) according to our automatic decision scheme. In case there are doubts on the accuracy of the fluence delivery, a pre-treatment measurement will be performed before the next fraction. If this measurement confirms relevant errors in fluence delivery, replanning should be performed before the next treatment fraction is given. If, on the other hand, the pre-treatment verification does not indicate any clinically relevant deviations, in-vivo measurements will be repeated during the next fractions to verify whether the PDI differences might be related to systematic deviations in patient anatomy. Also in that case replanning should be considered. For fractionated treatments with a large number of fractions we consider this approach acceptable, since corrections to compensate for delivery errors can still be performed in the remaining treatment fractions. McDermott et al. [21] suggested a similar approach.

Several groups have investigated the use of CBCT scans to reconstruct delivered patient doses based on measured EPID images (‘dose of the day’) [25,26]. In the work of McDermott et al. [25] measured EPID images were back-projected to multiple planes in a (kilovoltage) CBCT, assuming a water-equivalent electron density. Van Elmpt et al. [26,27] acquired megavoltage CBCT scans with calibrated electron densities to derive the entrance energy fluence for each treatment beam by back-projection of measured PDIs. These fluences were then used for reconstruction of the dose distribution in the CBCT using a Monte Carlo dose algorithm. Our group applied the SIFT method to derive the entrance fluence from measured EPID images and then performed a dose reconstruction using kilovoltage CBCT scans with corrected Hounsfields units [28]. As replacement of the SIFT method, we can now also apply the entrance fluences, as derived from the CBCT-based in-vivo measurements, to reconstruct the delivered patient dose.

The results of this study demonstrate for prostate cancer patients that high accuracy in-vivo verification of the delivered 3D patient dose based on CBCT might be complicated by intra-fraction anatomy variations. Likely, this can be alleviated by the combining analyses performed in 2D for many fractions. Investigations on the combination of several fractions in back projected EPID measured fluencies to estimate delivered 3D patients dose distributions, is a topic for further research that is important for so-called dose-guided radiotherapy.

In this paper we have demonstrated that in-room CBCT and in-vivo measured EPID images can be used for high accuracy in-vivo IMRT fluence verification for prostate cancer patients that are known for intra-fraction patient anatomy changes [20]. For other sites, the impact of such effects on the accuracy of CBCT-based fluence verification has to be investigated. The developed technology may also be applied for fluence verification of VMAT, stereotactic treatment, and 3DCRT. Accuracy assessment for these applications is a topic for further study.

## Conclusion

In-room acquired CBCT scans can be used for high accuracy IMRT fluence verification for prostate cancer, based on in-vivo measured EPID images. Combination of γ results for the first 2 fractions can largely compensate for small accuracy reductions with respect to pre-treatment verification, related to intra-fraction motion and anatomy changes. Compared to fluence verification based on the pCT-scan, accuracy of verification using in-room CBCT scans is clearly enhanced.

## Consent

In this study new tools to verify the accuracy of IMRT delivery using an EPID were developed and evaluated. This work did not affect patient’s treatment. Digital information, already routinely acquired for patient-specific QA, was used, i.e., for patients no additional investigations or measurements were required. For these reasons, according to the Ethics Committee of our hospital, no ethical approval was required for this study. Providing that all patient-related information was anonymized prior to presentation and publication, informed consent from the patients was not needed either.

## Competing interests

The authors declare that they have no competing interests.

## Authors’ contributions

AA was responsible for setting up this study; he contributed to the collection, analysis, interpretation of the data. He was also responsible for drafting the article. RC contributed to writing part of the software used in this study and he assisted in performing the phantom measurements. MD was the daily supervisor of the work. He assisted in setting up the research work, analyzing the results and revising the manuscript critically. BH was involved as general advisor and was responsible for the final results of this work. All authors read and approved the final manuscript.
